# R-Spondin 3 Regulates Dorsoventral and Anteroposterior Patterning by Antagonizing Wnt/β-Catenin Signaling in Zebrafish Embryos

**DOI:** 10.1371/journal.pone.0099514

**Published:** 2014-06-11

**Authors:** Xiaozhi Rong, Chen Chen, Pin Zhou, Yumei Zhou, Yun Li, Ling Lu, Yunzhang Liu, Jianfeng Zhou, Cunming Duan

**Affiliations:** 1 Key Laboratory of Marine Drugs (Ocean University of China), Chinese Ministry of Education, School of Medicine and Pharmacy, Ocean University of China, Qingdao, Shandong, China; 2 Department of Molecular, Cellular, and Developmental Biology, University of Michigan, Ann Arbor, Michigan, United States of America; Institute of Cellular and Organismic Biology, Taiwan

## Abstract

The Wnt/β-catenin or canonical Wnt signaling pathway plays fundamental roles in early development and in maintaining adult tissue homeostasis. R-spondin 3 (Rspo3) is a secreted protein that has been implicated in activating the Wnt/β-catenin signaling in amphibians and mammals. Here we report that zebrafish Rspo3 plays a negative role in regulating the zygotic Wnt/β-catenin signaling. Zebrafish Rspo3 has a unique domain structure. It contains a third furin-like (FU3) domain. This FU3 is present in other four ray-finned fish species studied but not in elephant shark. In zebrafish, *rspo3* mRNA is maternally deposited and has a ubiquitous expression in early embryonic stages. After 12 hpf, its expression becomes tissue-specific. Forced expression of *rspo3* promotes dorsoanterior patterning and increases the expression of dorsal and anterior marker genes. Knockdown of *rspo3* increases ventral-posterior development and stimulates ventral and posterior marker genes expression. Forced expression of *rspo3* abolishes exogenous Wnt3a action and reduces the endogenous Wnt signaling activity. Knockdown of *rspo3* results in increased Wnt/β-catenin signaling activity. Further analyses indicate that Rspo3 does not promote maternal Wnt signaling. Human RSPO3 has similar action when tested in zebrafish embryos. These results suggest that Rspo3 regulates dorsoventral and anteroposterior patterning by negatively regulating the zygotic Wnt/β-catenin signaling in zebrafish embryos.

## Introduction

The Wnt/β-catenin or canonical Wnt signaling pathway plays fundamental roles in early development and in maintaining adult tissue homeostasis in vertebrates [1]–[3]. In zebrafish embryos, the function of Wnt signaling is stage-specific. Maternal β-catenin, localized to the nucleus of dorsal marginal cells, is essential for the formation of the dorsal organizer before gastrulation [1]. Loss of maternal Wnt/β-catenin inhibits dorsal organizer formation and impairs the expression of genes required for dorsal organizer formation, such as *bozozok* (*boz*), *chordin* (*chd*), and *goosecoid* (*gsc*) [4]–[7]. Zygotic Wnt/β-catenin signaling, activated by Wnt ligands after mid-blastula transition, is required to initiate ventral cell fates to antagonize the organizer after gastrulation [8], [9]. Loss of Wnt8 in zebrafish embryos exhibits a significant expansion of the shield [10]. In addition, zygotic Wnt/β-catenin signaling is also involved in anterior-posterior neuroectoderm patterning [11]–[13]. Loss of Wnt8 in zebrafish embryos or overexpression of Wnt inhibitors such as *frzb*, *dickkopf*, and *Shisa* promotes head development [10], [14]–[16].

The activity of the canonical Wnt signaling pathway is regulated by a number of secreted proteins, including DKK1 and R-spondin (RSPO) proteins [14], [17]–[24]. While DKK1 inhibits the canonical Wnt signaling, RSPO3, a member of the RSPO family, has been suggested to activate Wnt signaling activity in mice and *Xenopus* [22], [25]. Mammalian and amphibian RSPO3/Rspo3s contain two furin-like cysteine-rich (FU) domains near the N-terminus, a thrombospondin type I (TSP1) domain in the central region, and a positively charged C-terminal region [26], [27]. Knockdown of *rspo3* causes ventral edema and vascular defects in *Xenopus* [28]. *Rspo3*-null mice suffer from severe vascular defects and are embryonic lethal [28], [29]. In *Xenopus*, the effects of Rspo3 gain- and loss-of-function on expression of hematopoietic markers were similar to that of Wnt8 [28]. Loss of Rspo3 in mice resulted in reduced Wnt reporter activity [28]. These results have lead to the notion that Rspo3 promotes Wnt signaling activity in mice and *Xenopus*. To date, the structure and function of RSPO3/Rspo3 in other vertebrates such as fish have not been reported.

In this study, we have determined the structure of the Rspo3 in zebrafish and several ray-finned fish species and discovered that all ray-finned fish Rspo3 orthologs contain three furin-like (FU) domains. In contrast, a cartilagous fish, elephant shark Rspo3 has two FU domains, like mammalian RSPO3/Rspo3. Using zebrafish as an experimental model, we investigated the developmental role of Rspo3. Unexpectedly, our results suggest that Rspo3 regulates dorsoventral and anteroposterior patterning in zebrafish embryos by negatively regulating the zygotic Wnt/β-catenin signaling.

## Materials and Methods

### Chemicals and Reagents

Restriction enzymes were purchased from New England BioLabs (Ipswich, MA, USA). Oligo(dT)_18_ was purchased from Sangon Biotech (Shanghai, China). iQ SYBR Green Supermix was purchased from Bio-Rad (Hercules, CA, USA). DIG-UTP and Anti-Digoxigenin-AP were purchased from Roche (Indianapolis, IN, USA). PCR primers were synthesized by Sangon Biotech and their sequences are shown in Table S1.

### Experimental Animals

Wild-type zebrafish (*Danio rerio*, Tübingen and AB strains) were maintained on a 14-h light/10-h dark cycle at 28.5°C and fed twice daily. Embryos obtained by natural cross were kept in embryo rearing solution and staged according to standard methods [30]. In some experiments, 2-phenylthiourea [0.003% (w/v)] was added to prevent embryonic pigment formation. Animal manipulation was performed under tricaine for anesthesia of fish, and all efforts were made to minimize suffering. All experimental protocols were approved by and conducted in accordance with the Ethical Committee of Experimental Animal Care, Ocean University of China (Permit Number: 11001).

### Molecular Cloning and Sequence Analysis

The full-length cDNA sequence of zebrafish *rspo3* was determined by 5′- and 3′- rapid amplification of cDNA ends (RACE) using the SMART RACE cDNA Amplification Kit (Clontech Laboratories, Mountain View, CA, USA) following the manufacturer’s instructions. The sequence of spotted gar, medaka, fugu, and stickleback Rspo3 were retrieved from Ensembl (www.ensembl.org) and that of elephant shark Rspo3 from http://esharkgenome.imcb.a-star.edu.sg/. The amino acid sequence alignment was performed using the GeneDoc software (Free Software Foundation). The phylogenetic tree was constructed using the Neighbor-Joining method with MEGA 4 software (The Biodesign Institute, Tempe, AZ, USA). The bootstrap analyses were run on 1,000 replicates with amino acid substitutions of the JTT model. The genomic structure of the elephant shark, spotted gar, zebrafish, medaka, fugu, and stickleback *rspo3* gene was obtained using the Blat program (http://genome.ucsc.edu/cgi-bin/hgBlat) and GENSCAN (http://genes.mit.edu/GENSCAN.html).

### Plasmid Construction

For functional analysis, cDNA encoding the zebrafish *rspo3* open reading frame (ORF) was amplified by reverse transcription-polymerase chain reaction (RT-PCR) using KOD plus DNA polymerase (TOYOBO, Shanghai, China) and cloned into the pCS2+ enhanced green fluorescent protein (EGFP) expression vector.

### RT-PCR and Whole Mount *in situ* Hybridization

Total RNA was isolated from zebrafish embryos using TRIzol reagent (Invitrogen, Carlsbad, CA, USA) and then reverse transcribed into first-strand cDNA using M-MLV (Promega, Madison, WI, USA) with Oligo(dT)_18_ as primer. RT-PCR was carried out using premix Taq DNA polymerase (Takara, Dalian, China). Quantitative real-time RT-PCR (RT-qPCR) was performed in an iCycler iQ Multicolor real-time PCR detection system (Bio-Rad Laboratories). Samples from 3 independent experiments were collected and each sample was measured in duplicate. The levels of mRNA of the gene of interest were calculated using the 2^−ΔΔCt^ method and normalized by *β-actin* mRNA levels [31].

The plasmid containing the *rspo3* partial ORF and 3′ untranslated region (UTR) was used to generate sense and antisense riboprobes using DIG RNA labeling mix (Roche, Indianapolis, IN, USA) following standard procedures. The specificity of the riboprobes was verified using dot-blot assay. *In situ* hybridization was performed as described previously [32].

### Morpholinos, mRNA Synthesis, and Microinjection

To knockdown *rspo3*, two translation-blocking morpholino oligonucleotides (MOs) targeting *rspo3,* MO1 (5′- TGGAGATCAGTTGCAATTGCATAGT -3′) and MO2 (5′- TATCGCACTGTGATGTGTGCAATAC -3′), were designed and purchased from Gene Tools (Philomath, OR, USA). A standard control MO from Gene Tools was used as the control. All MOs were dissolved in 1×Danieau buffer (58 mM NaCl, 0.7 mM KCl, 0.4 mM MgSO_4_, 0.6 mM Ca(NO_3_)_2_, 5 mM HEPES, pH 7.6) and diluted to the desired concentration. Capped mRNA was synthesized using mMESSAGE mMACHINE Kit (Ambion, Austin, TX, USA). Diluted MOs and/or mRNA were injected into one- to two-cell zebrafish embryos. A GFP reporter plasmid 5′-UTR-GFP, which contains the 5′ UTR and partial ORF (−102–72 bp) of zebrafish *rspo3,* was constructed and used to examine the efficiency of the MOs.

### Luciferase Assays

Luciferase assays were performed as reported previously [33]. Briefly, one- to two-cell stage embryos were injected with morpholinos and/or mRNA plus 100 pg Topflash DNA and 20 pg *Renilla* plasmid DNA, and raised to the shield stage. Two independent groups of embryos (each with more than 15 embryos) were lysed. The luciferase reporter assay was performed using a Dual-Luciferase Assay Kit (Promega). The Topflash luciferase activity was normalized by the *Renilla* luciferase activity.

### Statistical Analysis

Data are presented as Means+S.E. Differences among groups were analyzed by one-way ANOVA followed by Tukey’s Multiple Comparison Test, Chi-Square test or unpaired t-Test using GraphPad Prism version 5.01 (San Diego, CA, USA). Statistical significance was accepted at *P*<0.05 or smaller *p* values.

## Results

### Zebrafish and Other Bony Fish *rspo3* Orthologs have Unique Structural Features

**Figure 1:**
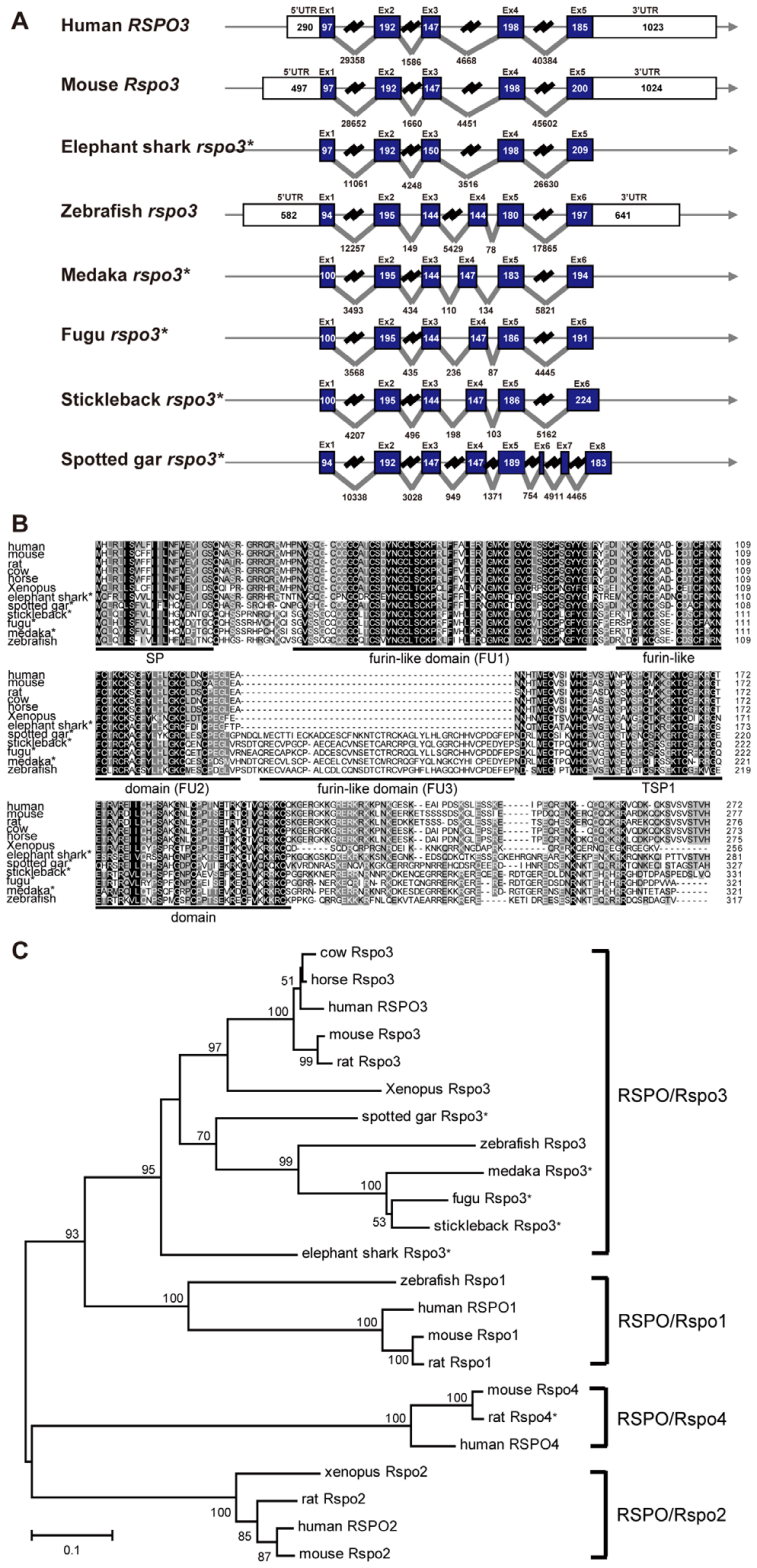
Gene structure, amino acid sequence, and phylogenetic analysis of zebrafish and other vertebrate Rspo3 orthologs. (**A**) Comparison of human, mouse, elephant shark, zebrafish, medaka, fugu, stickleback, and spotted gar *RSPO3/rspo3* gene structure. Exons are shown as boxes (filled box, protein coding region; open box, UTR). Introns are shown as lines. (**B**) Amino acid sequence alignment of human, mouse, rat, cow, horse, *Xenopus*, elephant shark, spotted gar, stickleback, fugu, medaka, and zebrafish RSPO/Rspo3. The identical amino acids are in black and similar amino acids are in grey. Protein domains of zebrafish Rspo3 are shown using black lines, and the domain names are given. (**C**) Phylogenetic analysis of the RSPO/Rspo family using the Neighbor-Joining (NJ) method. The length of branches represents the genetic distances, and numbers on nodes are bootstrap percentages to indicate the two clades as sisters. The accession numbers are as follows: human RSPO1 NP_001033722.1, mouse Rspo1 NP_619624.2, rat Rspo1 NP_001101450.1, zebrafish Rspo1 NP_001002352.1, human RSPO2 NP_848660.3, mouse Rspo2 NP_766403.1, rat Rspo2 NP_001124047.1, *Xenopus* Rspo2 NP_001088999.1, human RSPO3 NP_116173.2, mouse Rspo3 NP_082627.3, rat Rspo3 NP_001094460.1, cow Rspo3 NP_001069502.1, horse Rspo3 NP_001103152.1, *Xenopus* Rspo3 NP_001123245.1, medaka Rspo3 ENSORLP00000007233, fugu Rspo3 ENSTRUP00000009202, stickleback *rspo3* ENSGACG00000006080, elephant shark Rspo3 SINCAMP00000010032, spotted gar Rspo3 ENSLOCP00000020398, human RSPO4 NP_001025042.2, mouse Rspo4 NP_001035779.1, rat Rspo4 XP_006235323.1. *,Ensembl or GenBank predicted sequence.

By searching public databases and performing 5′- and 3′-RACE experiments, we cloned the full-length zebrafish *rspo3* cDNA and determined its gene structure (zgc: 162040). While human and mouse *RSPO3*/*Rspo3* genes have 5 exons, zebrafish *rspo3* contains 6 exons (Fig. 1A). Zebrafish *rspo3* encodes a protein that shares high sequence identities to known RSPO3s. The overall sequence identities of zebrafish Rspo3 to its human and *Xenopus* orthologs are 45% and 48% (Fig. 1B). Phylogenetic analyses suggested that the zebrafish Rspo3 is a *bona fide* Rspo3 (Fig. 1C). Zebrafish Rspo3 has an overall domain arrangement similar to its mammalian and amphibian orthologs (Fig. 1B). Unlike their mammalian and *Xenopus* counterparts, however, zebrafish Rspo3 has three FU domains. We also determined the *rspo3* gene and protein structure in medaka, fugu, and stickleback. They all likewise have an additional FU3 domain encoded by an extra exon (Fig. 1A and 1B). Searching these teleost genomes suggested that they contain only one *rspo3* gene. To determine when the FU3 domain was evolved, we obtained the *rspo3* sequence from the spotted gar (a non-teleost bony fish) and elephant shark (a cartilaginous fish). As shown in Fig. 1B, spotted gar Rspo3 also has three FU domains. In contrast, elephant shark Rspo3 has two FU domains. These findings suggest that the third FU domain was gained in the ray-finned fish lineage.

### Developmental Expression Pattern of Zebrafish *rspo3*

**Figure 2:**
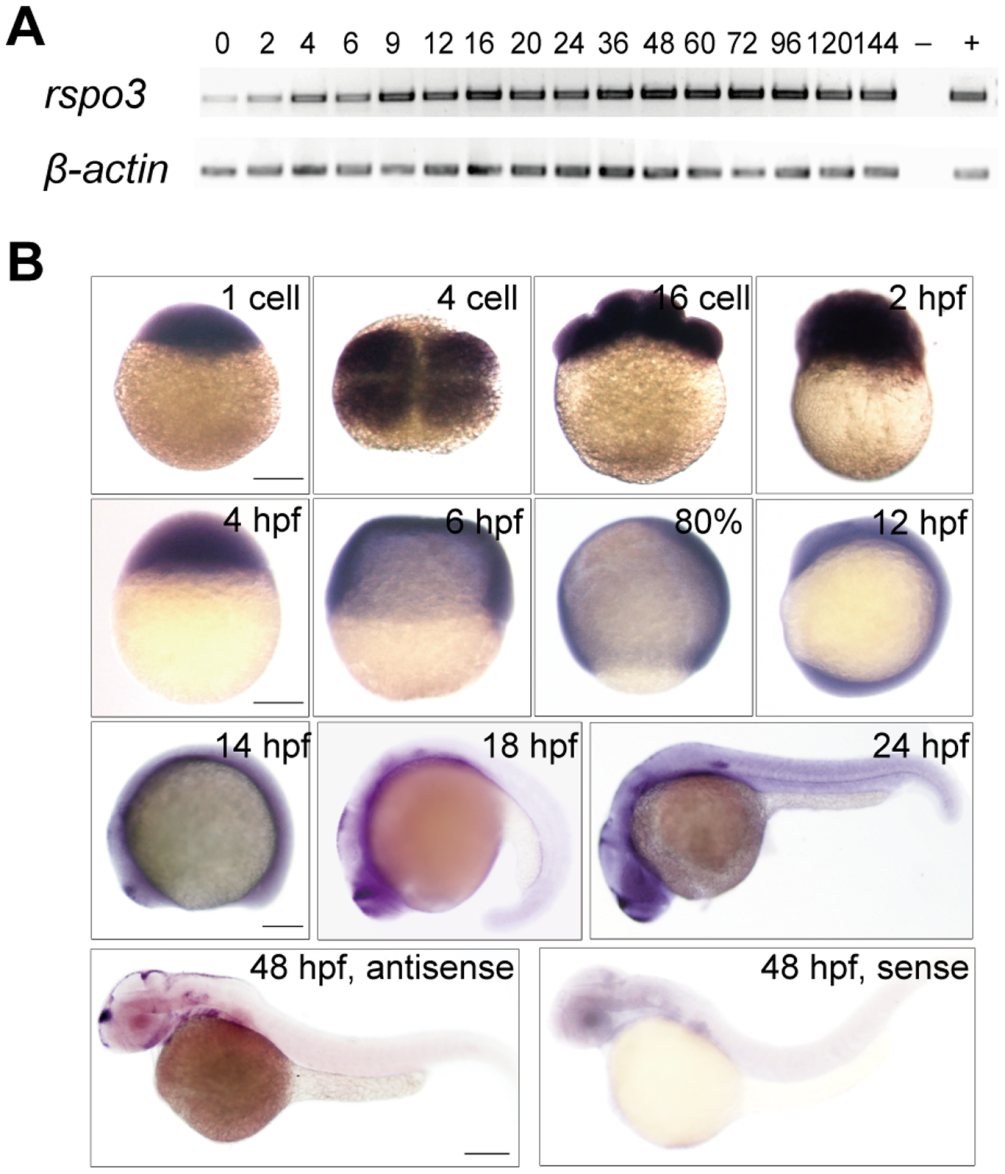
The spatiotemporal expression pattern of zebrafish *rspo3*. (**A**) RT-PCR analysis of zebrafish *rspo3* mRNA at the indicated embryonic stages. Numbers indicate different developmental stages as hours post fertilization (hpf). *β-actin* as internal control. −, negative control; +, positive control. (**B**) Whole-mount *in situ* hybridization analysis of zebrafish *rpso3* mRNA at the indicated stages. All panels are dorsal, top, or lateral views with animal pole up or anterior to the left. Scale bars = 200 µm.

RT-PCR analysis results showed that zebrafish *rspo3* mRNA was expressed in all of the examined stages ranging from 1-cell to 144 hours post fertilization (hpf, Fig. 2A). Whole-mount *in situ* hybridization results indicated that the *rspo3* transcript was expressed in a ubiquitous manner from the 1-cell stage to 12 hpf. At 14 hpf and 18 hpf, the *rspo3* transcript began to be highly expressed in telencephalon, metencephalon, cephalic floor plate, and otic vesicle. At 24 hpf, strong *rspo3* mRNA signal was observed in telencephalon, diencephalon, metencephalon, rhombencephalon, cephalic floor plate, lateral line precordium, and hypochord. At 48 hpf, strong signals were detected in telencephalon, diencephalon, metencephalon, rhombencephalon, lateral line primordium, branchial arches, palatoquadrate, and hypochord (Fig. 2B).

### Overexpression and Knockdown Studies Reveal that *rspo3* Regulates Dorsoventral and Anteroposterior Patterning in Zebrafish

**Figure 3:**
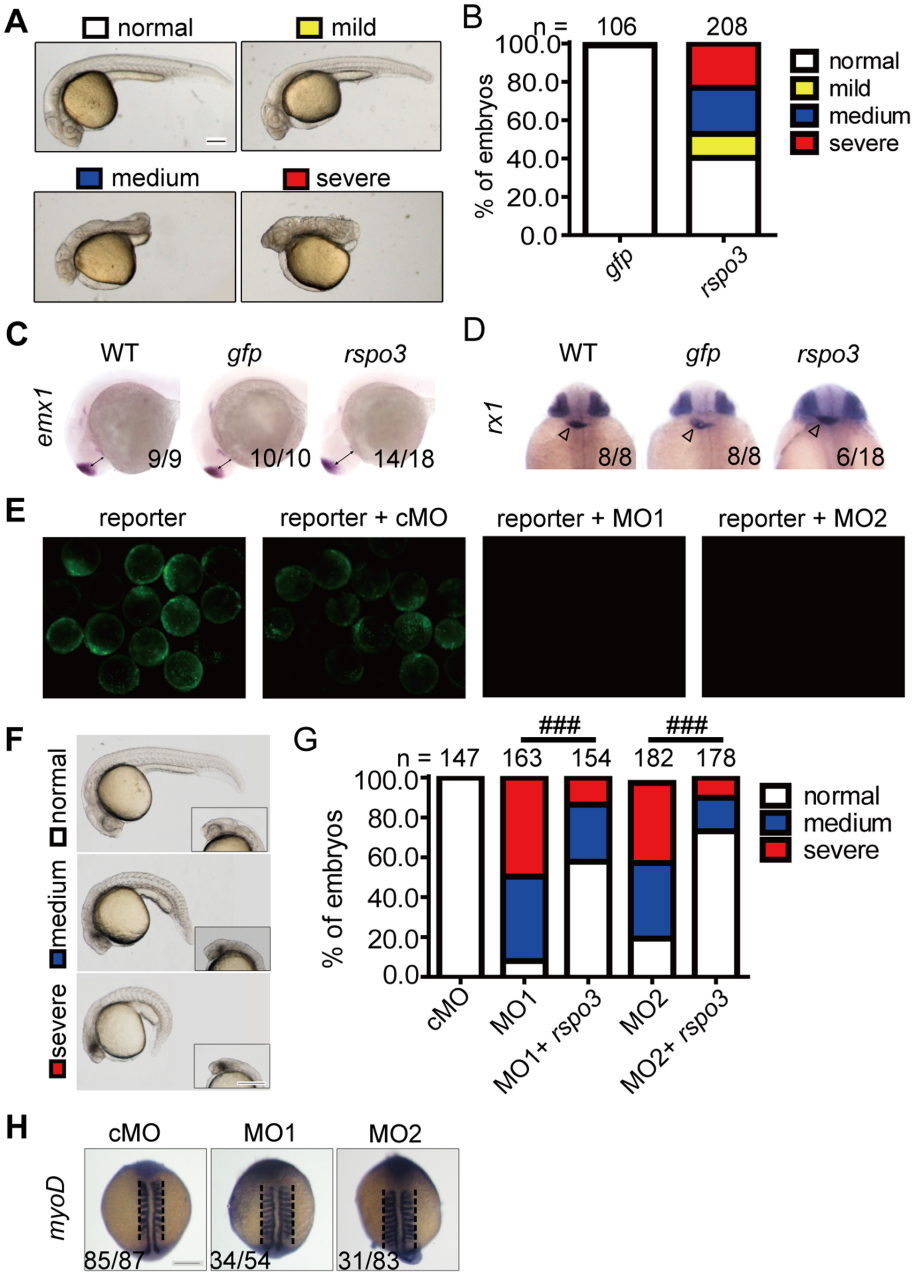
Effects of *rspo3* overexpression and knockdown in zebrafish embryos. (**A**) Classification of phenotypes caused by forced expression of *rspo3*. One-cell stage embryos were injected with 600 pg *rspo3* mRNA. Embryos were raised to 24 hpf and examined. Lateral views with anterior to the left. Scale bar = 200 µm. (**B**) The percentages of embryos in each category as shown in (A). The results are from three independent experiments and the total embryo numbers are given at the top. (**C, D**) Expression patterns of the indicated marker genes in wild-type (WT) embryos or embryos injected with 600 pg *gfp* mRNA or *rspo3* mRNA. Embryos were analyzed at 24 hpf by *in situ* hybridization. Lateral view with anterior to the left (C) and dorsal view with anterior up (D) are shown, and the frequency of embryos with the indicated patterns is shown in the bottom right in each panel. Double arrow lines in C show the distance from the telencephalon to the yolk. Blank arrow heads in D indicate the heart labeled by *nkx2.5* mRNA. (E) Effectiveness and specificity of MOs used. Fluorescent micrographs of zebrafish embryos at 12 hpf injected with the *rspo3* 5′-UTR reporter plasmid alone (100 pg), the reporter plasmid DNA with control MO (4 ng), *rspo3* targeting MO1 (4 ng) or *rspo3* targeting MO2 (8 ng), respectively. (**F**) Classifications of phenotypes caused by morpholino-mediated knockdown of *rspo3*. Representative views of zebrafish embryos at 24 hpf injected with 8 ng control MO (cMO), 4 ng (MO1) or 8 ng (MO2) *rspo3* targeting MO, and 4 ng MO1 or 8 ng MO2 plus 20 pg *rspo3* mRNA (MO+*rspo3*). Lateral views with anterior to the left. The amplified head region of each embryo is shown in right corner insert. Scale bar = 200 µm. (**G**) The percentages of embryos in each category as shown in (F). The results are from three independent experiments and the total embryo numbers are given at the top. ### *P*<0.0001, Chi-Square test. (**H**) Expression patterns of the indicated marker genes in embryos injected with 8 ng cMO, 4 ng *rspo3* MO1, or 8 ng MO2. Embryos were analyzed at 14 hpf by *in situ* hybridization. Dorsal view with anterior to the top is shown, and the frequency of embryos with the indicated patterns is shown in the bottom left corner of each panel. The blank dash lines show the extension of the marker expression. Scale bar = 200 µm. Next, knockdown experiments were carried out using two independent translation- blocking antisense MOs. The efficacy of these *rspo3* targeting MOs was verified by co-injecting an *rspo3* 5′-UTR-GFP expression construct. Both MO1 and MO2 blocked the reporter GFP expression (Fig. 3E). Knockdown of *rspo3* by either MO1 or MO2 resulted in an increase in the number of embryos displaying enhanced ventral-posterior phenotypes (Fig. 3F and 3G). In addition, knockdown of *rspo3* resulted in lateral expansion of somites as indicated by *myoD* mRNA expression at 14 hpf (Fig. 3H). The abnormal embryos were classified into medium and severe groups (Fig. 3F and 3G). Embryos in the medium group exhibited smaller eyes, slightly reduced head, and curved body axis (Fig. 3F). Embryos in the severe group exhibited smaller eyes, reduced brain, and shorter and curved body axis (Fig. 3F). 50% and 42% of the MO1-injected embryos were in the severe and medium group (Fig. 3G). Likewise, 40% and 38% of the MO2-injected embryos were in the severe and medium group (Fig. 3G). Importantly, co-injection of *rspo3* mRNA with MO1 or MO2 markedly reduced the MO-induced abnormal phenotypes from ∼90% to ∼40% (MO1) and ∼80% to ∼25% (MO2), respectively (*p*<0.0001) (Fig. 3G).

To investigate the developmental role(s) of zebrafish Rspo3, we performed mRNA injection experiments. Injection of *rspo3* capped mRNA increased dorsoanterior phenotypes (Fig. 3A and 3B). These abnormal embryos were classified morphologically into three groups: mild, medium, and severe (Fig. 3A). Embryos in the mild group exhibited shortened body axis (Fig. 3A). Embryos in the medium group exhibited truncated posterior body axis and curved tail (Fig. 3A). Embryos in the severe group lacked the posterior body axis (Fig. 3A). As shown in Fig. 3B, injection of *rspo3* mRNA resulted in a 23%, 24%, and 12% increase in the severe, medium, and mild group. We next performed *in situ* hybridization with *emx1* (labeling forebrain) and *rx1* (labeling retina). As shown in Fig. 3C and 3D, injection of *rspo3* mRNA resulted in 78% and 33% embryos with enlarged brain and eyes, respectively.

Next, knockdown experiments were carried out using two independent translation- blocking antisense MOs. The efficacy of these *rspo3* targeting MOs was verified by co-injecting an *rspo3* 5’-UTR-GFP expression construct. Both MO1 and MO2 blocked the reporter GFP expression (Fig. 3E). Knockdown of *rspo3* by either MO1 or MO2 resulted in an increase in the number of embryos displaying enhanced ventral-posterior phenotypes (Fig. 3F and 3G). In addition, knockdown of *rspo3* resulted in lateral expansion of somites as indicated by *myoD* mRNA expression at 14 hpf (Fig. 3H). The abnormal embryos were classified into medium and severe groups (Fig. 3F and 3G). Embryos in the medium group exhibited smaller eyes, slightly reduced head, and curved body axis (Fig. 3F). Embryos in the severe group exhibited smaller eyes, reduced brain, and shorter and curved body axis (Fig. 3F). 50% and 42% of the MO1-injected embryos were in the severe and medium group (Fig. 3G). Likewise, 40% and 38% of the MO2-injected embryos were in the severe and medium group (Fig. 3G). Importantly, co-injection of *rspo3* mRNA with MO1 or MO2 markedly reduced the MO-induced abnormal phenotypes from ∼90% to ∼40% (MO1) and ∼80% to ∼25% (MO2), respectively (*p*<0.0001) (Fig. 3G).

**Figure 4:**
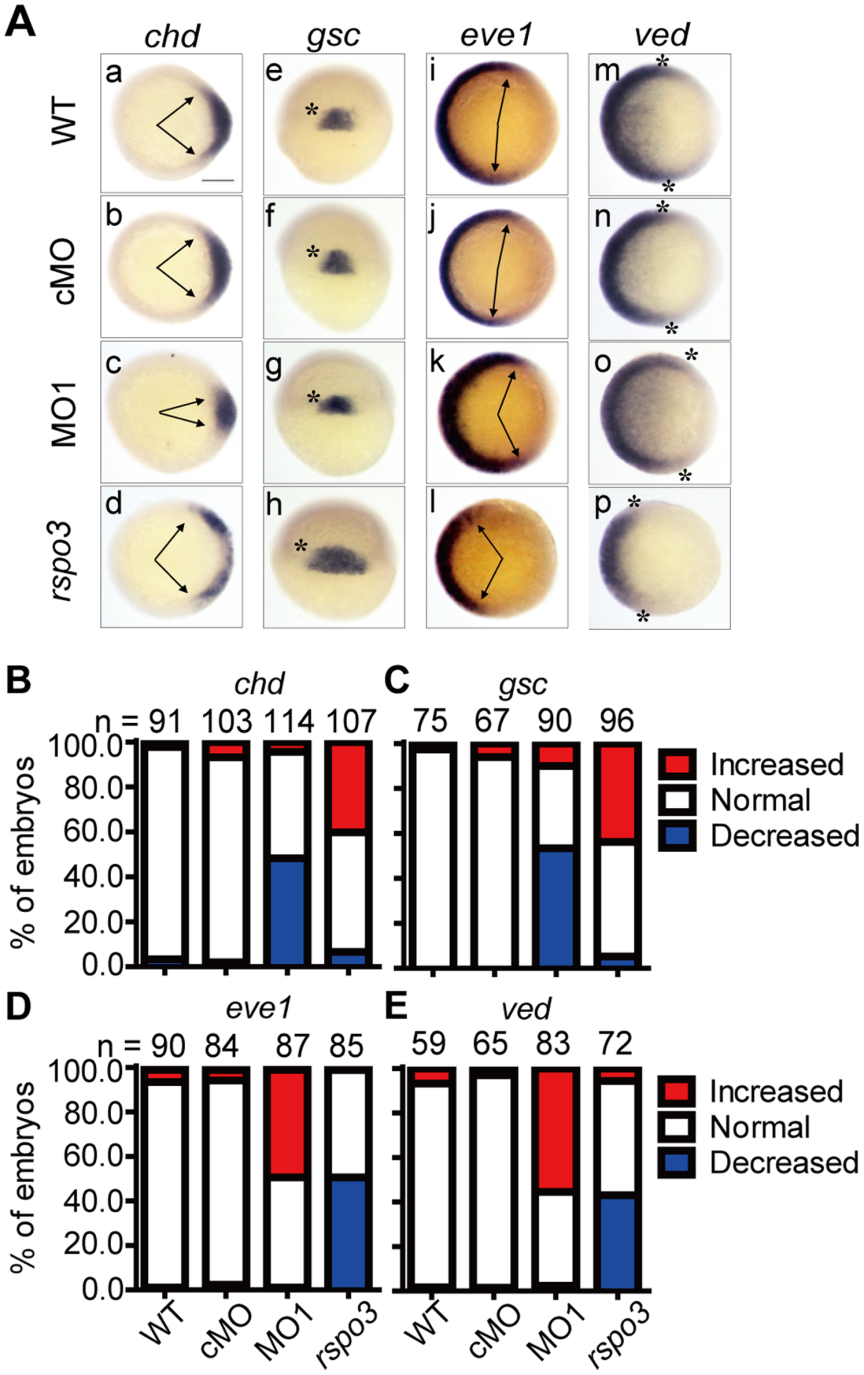
Zebrafish Rspo3 regulates dorsoventral patterning. (**A**) Expression patterns of the indicated marker genes in wild-type (WT) embryos or embryos injected with 4 ng control MO (cMO), 4 ng *rspo3* targeting MO1 or 600 pg *rspo3* mRNA, respectively. Embryos were analyzed at the shield stage by *in situ* hybridization using the indicated probes. Panels a–d and i-p are top views with animal pole up and dorsal to the right; panels e–h are lateral and dorsal views with animal pole up. Arrows indicate the width of the expression domains; asterisks indicate the edges of the expression domains. Percentages of embryos in each category were calculated and are shown in **B** (*chd*), **C** (*gsc*), **D** *(eve1)* and **E** (*ved*). The total embryo numbers from three independent experiments are shown on the top of each bar. Scale bar = 200 µm.

Next, we performed *in situ* hybridization using a number of dorsoventral marker genes. The dorsal markers *chordin* (*chd*) and *gooscoid* (*gsc*) were expressed on the dorsal embryonic shield in wild-type and control MO (cMO)-injected embryos (Fig. 4A–C). Their expression domains were reduced in MO1-injected embryos (Fig. 4A–C). In contrast, the expression domains of *even-skipped-1* (*eve1*) and *ventral edema* (*ved*), two ventral mesoderm marker genes, were increased in the morphants (Fig. 4A, 4D, and 4E). Forced expression of *rspo3*, on the other hand, increased *chd* and *gsc* mRNA expression domains and reduced *eve1* and *ved* mRNA expression domains (Fig. 4A–E). These results suggest that Rspo3 promotes dorsoanterior development and inhibits ventral-posterior development in zebrafish.

### Rspo3 Does Not Promote Maternal Wnt Signaling in Zebrafish Embryos

**Figure 5:**
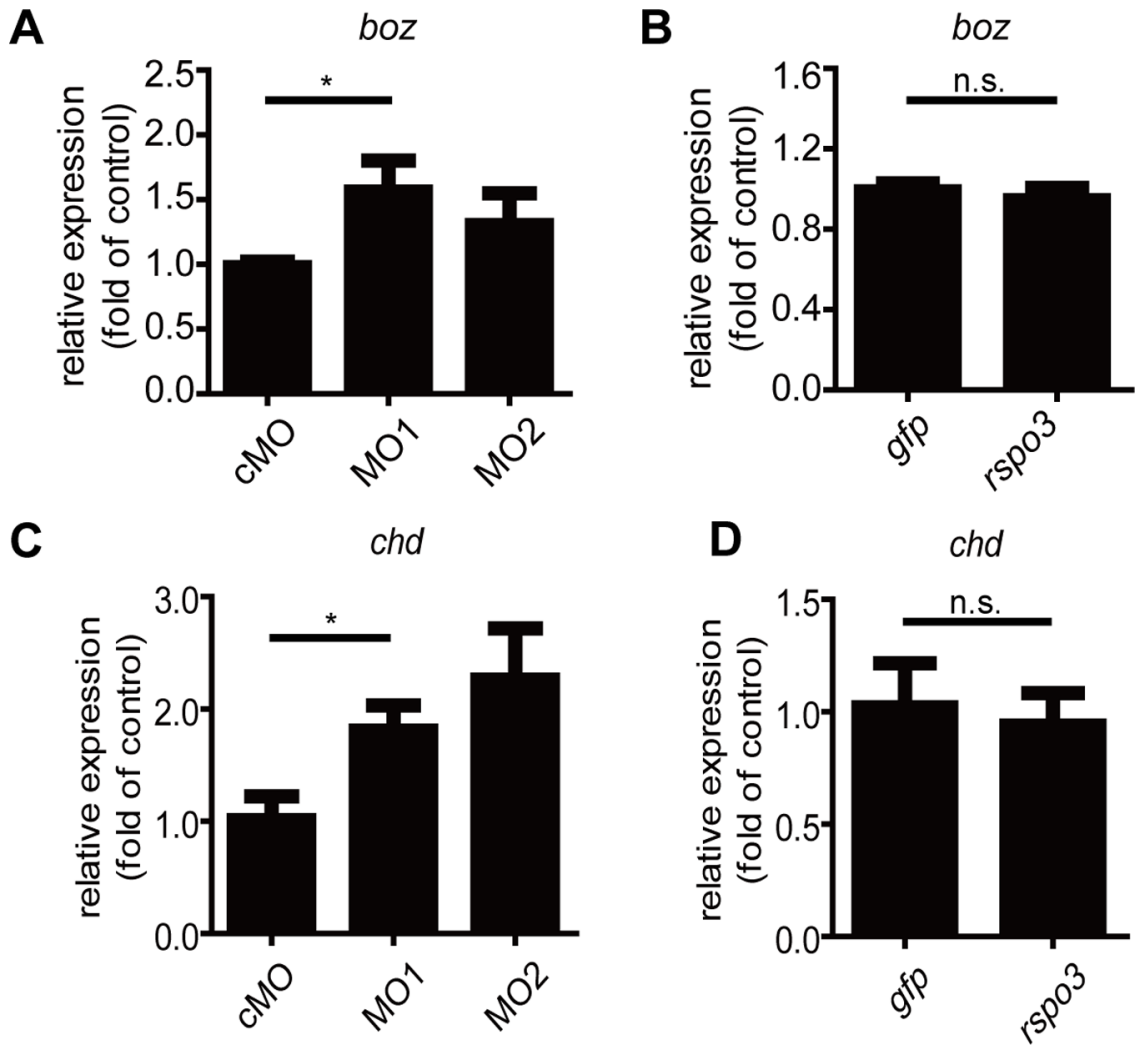
Rspo3 does not promote maternal Wnt/β-catenin signaling in zebrafish embryos. (**A, B**) Effect of *rspo3* knockdown (A) and overexpression (B) on the expression of *boz* mRNA. (**C, D**) Effect of *rspo3* knockdown (C) and overexpression (D) on the expression of *chd* mRNA. One-cell stage embryos were injected with 8 ng control MO (cMO), 4 ng MO1 and 8 ng MO2, respectively (A, C), or 600 pg *gfp* mRNA or *rspo3* mRNA (B, D). Injected embryos were analyzed by RT-qPCR at the sphere stage. Values are means ± S.E. (n = 3). *P<0.05; n.s., not significant, unpaired t-Test.

In zebrafish embryos, maternal and zygotic Wnt/β-catenin signaling manifests different biological effects [1], [3], [10], [34]–[36]. While maternal Wnt/β-catenin signaling in dorsal marginal cells establishes dorsal cell fates before gastrulation, zygotic Wnt/β-catenin signaling in ventrolateral regions initiates ventral cell fate after gastrulation [3], [4], [8], [37],[38]. Either promotion of the maternal Wnt activity or inhibition of the zygotic Wnt activity could result in the phenotypes observed above [4], [6], [39]. To distinguish these two possibilities, we first investigated the effects of Rspo3 on *bozozok* (*boz*) expression at the sphere stage when zygotic Wnt/β-catenin is not yet functional [5], [7]. In zebrafish, *boz* is a direct maternal Wnt signaling target gene [5], [7]. Knockdown of *rspo3* by MO1 or MO2 did not decrease *boz* mRNA levels (Fig. 5A). In fact, injection of MO1 even increased *boz* mRNA levels (Fig. 5A). Forced expression of *rspo3* did not change *boz* mRNA levels (Fig. 5B). We also examined the *chd* expression at the sphere stage. Expression of *chd* at the sphere stage is another read-out of maternal β-catenin activity [40]. Consistent with the *boz* results, knockdown of *rspo3* by MO1 actually increased *chd* mRNA levels (Fig. 5C). MO2 injection increased *chd* mRNA levels but the change did not reached statistical significance (Fig. 5C). Forced expression of *rspo3* did not change *chd* mRNA levels (Fig. 5D). Collectively, these results indicate that Rspo3 does not promote maternal Wnt/β-catenin activity.

### Rspo3 Inhibits Zygotic Wnt/β-catenin Signaling in Zebrafish Embryo

**Figure 6:**
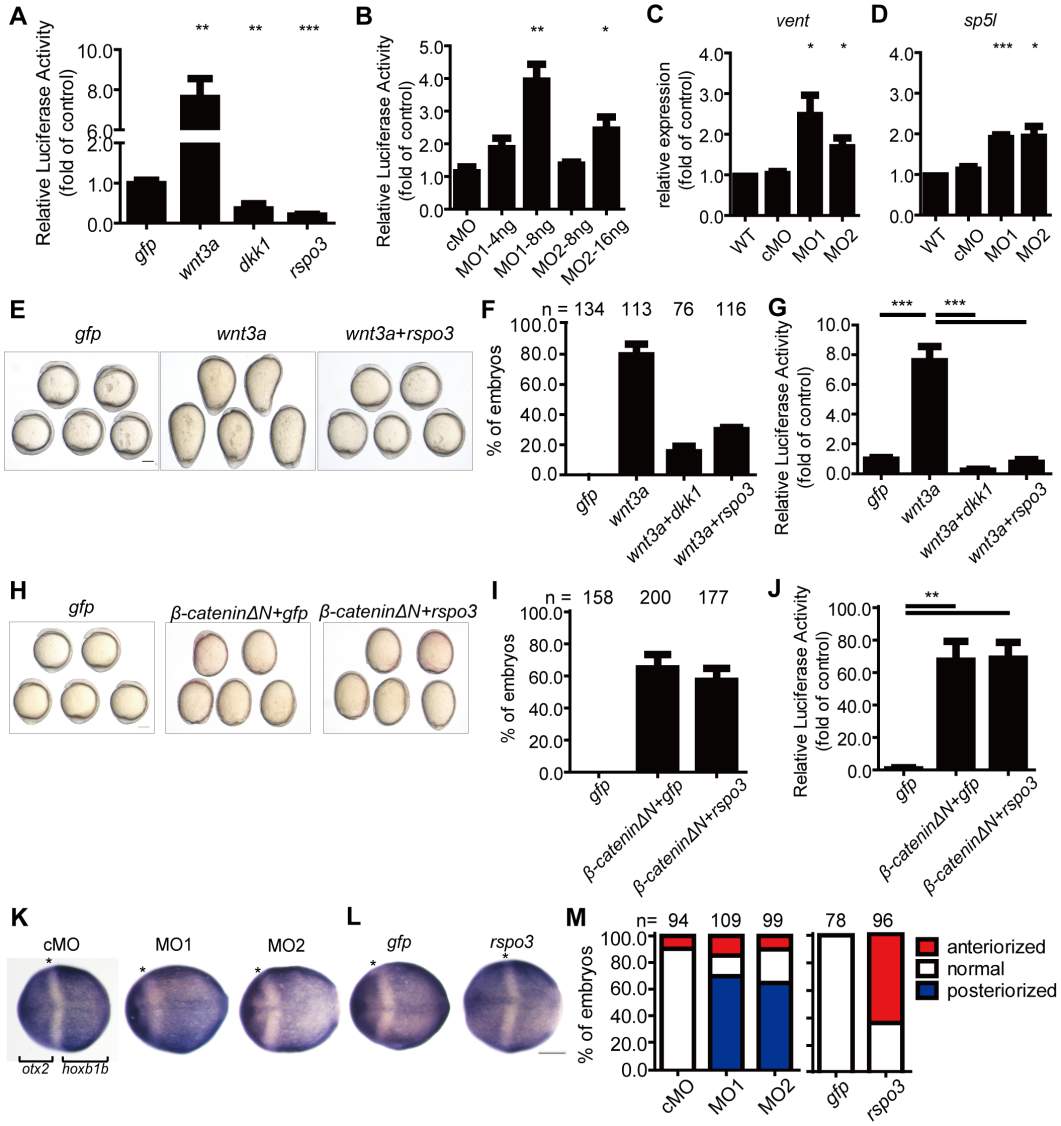
Rspo3 inhibits the zygotic Wnt/β-catenin signaling in zebrafish embryos. (**A**) Rspo3 inhibited Wnt/β-catenin reporter activities. One-cell stage embryos were injected with Topflash reporter DNA together with 600 pg *gfp* mRNA, 20 pg *wnt3a* mRNA, 200 pg *dkk1* mRNA, or 600 pg *rspo3* mRNA, respectively. Injected embryos were raised to the shield stage and the luciferase activity was measured. Values are means ± S.E. (n = 3). **, ***P<0.01 and 0.001 vs. the *gfp* group. (**B**) Knockdown of *rspo3* increased Wnt/β-catenin reporter activity. One-cell stage embryos were injected Topflash reporter DNA together with cMO (16 ng), MO1, or MO2, respectively. Injected embryos were raised to the shield stage and the luciferase activity was measured. (**C** and **D**) Knockdown of *rspo3* increased the expression levels of *vent* (C) and *sp5l* (D) mRNA. Embryos were injected with cMO (8 ng), MO1 (4 ng) or MO2 (8 ng) at the one-cell stage, and wild-type embryos were used as control. The *vent* and *sp5l* mRNA levels were measured by RT-qPCR at the 90% epiboly stage. Values are means ± S.E. (n = 3). *, **, ***P<0.05, 0.01, and 0.001 vs. the cMO group. (**E**) Rspo3 inhibited Wnt3a activity. Representative views of embryos. The pictures were taken at the 5-somite stage. (**F**) Quantitative results. The percentages of dorsalized embryos described in (E) were calculated and shown. The total embryo numbers are given at the top. Embryos were injected with 600 pg *gfp* mRNA, 20 pg *wnt3a* mRNA, 20 pg *wnt3a* mRNA plus 200 pg *dkk1*, or 600 pg *rspo3* mRNA. (**G**) Rspo3 inhibited Wnt3a-stimulated Topflash reporter activity. One-cell stage embryos were injected with Topflash DNA together with the indicated mRNA, the injected embryos were raised to the shield stage and luciferase activities were determined. Values are means ± S.E. (n = 3). ***P<0.0001, one-way ANOVA test. (**H**) Rspo3 cannot inhibit *β-cateninΔN* activity. Representative views of embryos injected with 600 pg *gfp* mRNA, 50 pg *β-cateninΔN* mRNA, or 50 pg *β-cateninΔN* plus 600 pg *rspo3* mRNA. The pictures were taken at the 5-somite stage. (**I, J**) Quantitative results (I) and luciferase activity assay (J). Values are means ± S.E. (n = 3). **P<0.01, one-way ANOVA test. (**K**) Knockdown of *rspo3* decreased the expression of *otx2* and increased the expression of *hoxb1b* at the 100% epiboly stage. (**L**) Overexpression of *rspo3* increased the expression of *otx2* and decreased the expression of *hoxb1b* at the 100% epiboly stage. Dorsal views with anterior to the left are shown. Asterisks indicate the edges of the expression domains (K, L). Scale bars = 200 µm. (**M**) The percentage of embryos were calculated and shown. The results are from three independent experiments, and the total embryo numbers are given at the top.

The phenotypes that resulted from *rspo3* overexpression resembled those caused by the loss of Wnt ligands and the overexpression of the Wnt inhibitor *dkk1* [36], [41]–[43]. The *rspo3* morphants resembled the *dkk1* morphant phenotypes [44]. We postulated that Rspo3 may negatively regulate the zygotic Wnt/β-catenin signaling. To test this hypothesis, we injected a Wnt reporter construct (Topflash), in which Wnt-responsive elements drive the expression of the luciferase reporter [45], together with Wnt3a, Dkk1, and Rspo3 mRNA. Forced expression of *wnt3a* in zebrafish embryos resulted in a robust increase in the Topflash reporter activity (Fig. 6A). Forced expression of *rspo3* or *dkk1* decreased the basal Topflash reporter activity (Fig. 6A), suggesting that Rspo3 plays an inhibitory role in regulating Wnt/β-catenin signaling. The role of endogenous Rspo3 in regulating the zygotic Wnt/β-catenin signaling was investigated next. Compared with the control group, knockdown of *rspo3* by either MO1 or MO2 increased the Wnt reporter activity in a dose-dependent manner at the shield stage (Fig. 6B). More importantly, knockdown of *rspo3* resulted in increased expressions of *vent* and *sp5l,* two direct Wnt target genes [46], [47], at the 90% epiboly stage (Fig. 6C and 6D).

Next, we performed co-injection experiments. Injection of *wnt3a* mRNA resulted in dorsalized phenotypes in more than 80% of the injected embryos at the 5-somite stage. Co-injection of *rspo3* mRNA with *wnt3a* mRNA reduced the percentages of dorsalized embryos to 30% (Fig. 6E and 6F). Similarly, co-injection of *dkk1* mRNA with *wnt3a* mRNA reduced the percentages of dorsalized embryos to 16% (Fig. 6F). Like Dkk1, co-expression of Rspo3 with Wnt3a abolished the Wnt3a-induced Topflash reporter activity (Fig. 6G), suggesting that Rspo3 inhibits the action of Wnt3a. Injection of *β-cateninΔN*, which encodes a constitutively active β-catenin lacking the first 45 N-terminal residues, led to dorsalized embryos at the 5-somite stage (Fig. 6H). Co-injection of *rspo3* with *β-cateninΔN* mRNA did not reduce the percentages of dorsalized embryos (Fig. 6I). Likewise, co-injection of *rspo3* with *β-cateninΔN* mRNA did not block Wnt reporter activity induced by *β-cateninΔN* (Fig. 6J). These results indicated that Rspo3 inhibits the zygotic Wnt/β-catenin signaling, likely acting at a step upstream of β-catenin.

One critical role of the zygotic Wnt/β-catenin signaling is to induce posterior neural fates [9], [10], [46], [48]. If Rspo3 indeed inhibits the zygotic Wnt/β-catenin signaling pathway in zebrafish, then knockdown of *rspo3* should increase posteriorization and forced expression of *rspo3* should lead to anteriorization. Indeed, knockdown of *rspo3* by either MO1 or MO2 resulted in a marked reduction in the expression of the anterior neuroectoderm marker *otx2* and a concomitant expansion of the posterior neural marker *hoxb1b* region (Fig. 6K and 6M). In contrast, forced expression of *rspo3* resulted in an expansion in the *otx2* mRNA expression and a reduction in the *hoxb1b* mRNA expression (Fig. 6L and 6M). These results support the notion that Rspo3 promotes dorsoanterior development by negatively regulating the Wnt/β-catenin signaling pathway in zebrafish.

**Figure 7:**
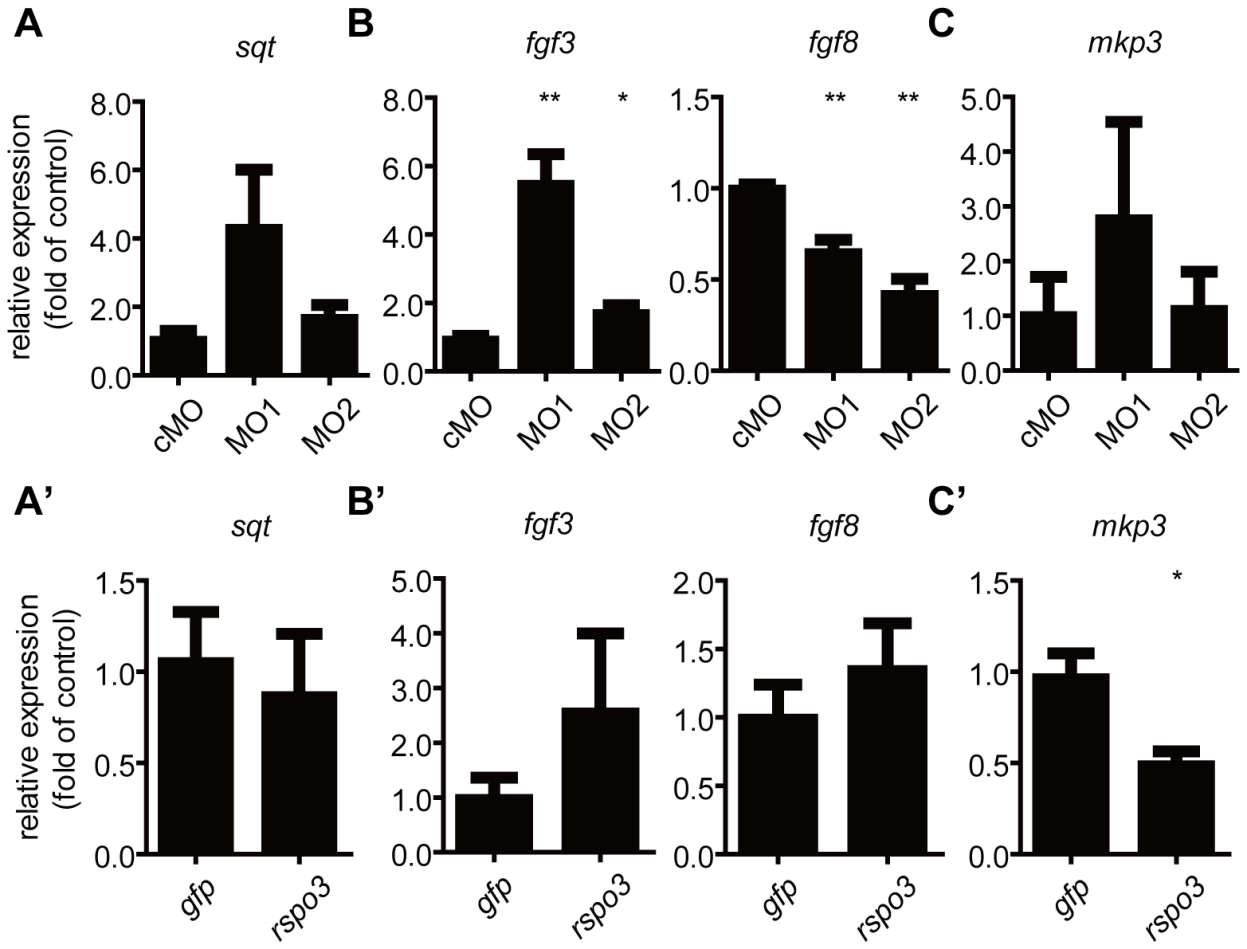
Effects of *rspo3* knockdown and forced expression on the expression of *fgf3*, *fgf8*, *mkp3*, and *sqt* mRNA. One-cell stage embryos were injected with cMO (8 ng), MO1 (4 ng), MO2 (8 ng), *gfp* mRNA (600 pg), or *rspo3* mRNA (600 pg), respectively. Injected embryos were raised to the 90% epiboly stage. The mRNA levels of *sqt*, *fgf3*, *fgf8*, and *mkp3* were measured by RT-qPCR, normalized by *β-actin* mRNA levels, and as shown. Values are means ± S.E. (n = 3). *, **P<0.05 and 0.01 vs. the *gfp* or cMO group.

In addition to Wnt/β-catenin signaling, Nodal and Fgf signaling have been shown to regulate dorsoventral patterning in zebrafish [49]–[51]. We examined the possible effects of the *rspo3* knockdown and overexpression on the expression of Nodal ligand *sqt* and Fgf ligands. Neither knockdown nor forced expression of *rspo3* changed the levels of *sqt* mRNA (Fig. 7A and 7A’). Knockdown of *rspo3* by both MO1 and MO2 increased the levels of *fgf3* mRNA, while both MO1 and MO2 injection decreased the levels of *fgf8* mRNA (Fig. 7B). However, forced expression of *rspo3* did not result in any significant changes in the levels of *fgf3* or *fgf8* mRNA (Fig. 7B’). Mkp3, which has similar expression domains with the Fgf ligands, also regulates dorsovental patterning in zebrafish embryos [52]. Knockdown of *rspo3* by either MO1 or MO2 did not affect expression of *mkp3* but forced expression of *rspo3* decreased *mkp3* mRNA levels (Fig. 7C and 7C’).

### Human RSPO3 Has a Similar Inhibitory Effect in Zebrafish Embryos

**Figure 8:**
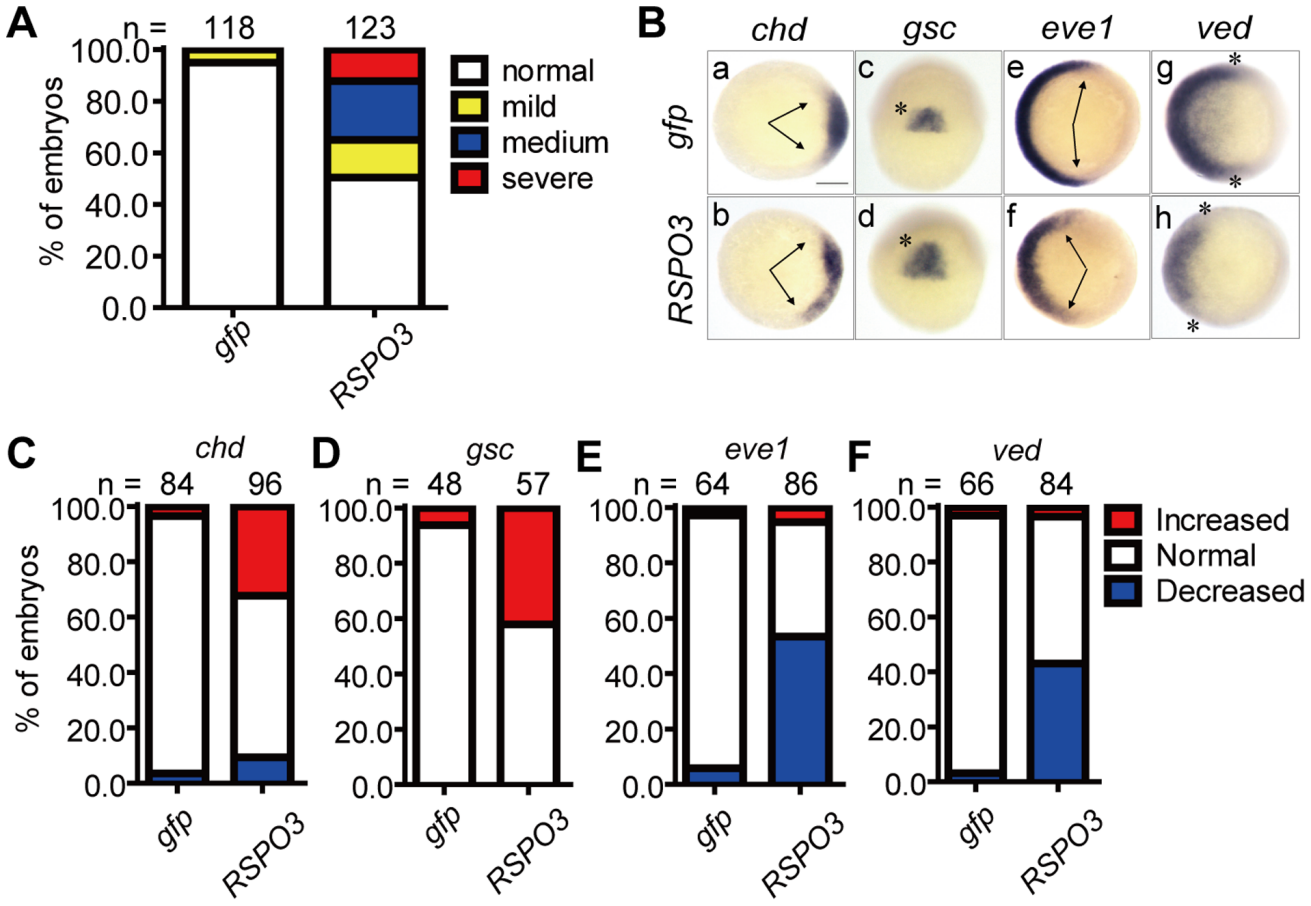
Effects of forced expression of human *RSPO3* in zebrafish embryos. (**A**) The phenotypes of embryos injected with 600 pg *gfp* or *RSPO3* mRNA were scored and presented following the criteria described in Fig. 3A. The results are from three independent experiments and the total embryo numbers are given at the top. (**B–F**) Human *RSPO3* alters the expression of the indicated genes in zebrafish embryos. Embryos injected with 600 pg *RSPO3* or *gfp* mRNA were analyzed by whole mount *in situ* hybridization at the shield stage using the indicated probes. Scale bar = 200 µm. Percentages of embryos in each category were calculated and shown in **C** (*chd*), **D** (*gsc*), **E** *(eve1)*, and **F** (*ved*). The total embryo numbers from three independent experiments are shown on the top of each bar.

To examine whether the inhibitory action of Rspo3 is due to the structural difference between teleost Rspo3 and mammalian RSPO3, we forced expression of human *RSPO3* in zebrafish embryos. Forced expression of human *RSPO3* resulted in similar phenotypes as zebrafish Rspo3 (Fig. 8A). Forced expression of human *RSPO3* also resulted in notable increases in *chd* and *gsc* expression domains (Fig. 8B a–d, 8C, and 8D) and decreased *eve1* and *ved* expression (Fig. 8B e–h, 8E, and 8F). These data suggest that human RSPO3 has inhibitory activity that is similar to that of zebrafish Rspo3.

## Discussion

In this study, we determined the structure of the Rspo3 protein and gene sequences in five ray-finned fish and a cartilagous fish. We mapped the spatial and temporal expression pattern of *rspo3* in zebrafish and determined its developmental role through overexpression and MO-mediated knockdown experiments. Our results suggest that bony fish Rspo3 has unique structural features and plays a previously unrecognized role in regulating dorsoventral and anterior-posterior patterning by antagonizing the zygotic Wnt/β-catenin signaling pathway in zebrafish embryos.

Both gain- and loss-of-function evidence supports our conclusion. Forced expression of *rspo3* promoted dorsoanterior patterning in zebrafish embryos. Forced expression of *rspo3* increased the expression of *chd* and *gsc* mRNA, while it reduced *eve1* and *ved* mRNA expression. Furthermore, forced expression of *rspo3* increased the expression of the anterior marker gene *otx2* but reduced the expression of posterior neural marker *hoxb1b*. Knockdown of *rspo3* had the opposite effects. These results suggest that Rspo3 promotes dorsoanterior patterning and inhibits ventral-posterior patterning in zebrafish.

A major finding made in this study is that Rspo3 functions as a negative regulator in the zygotic Wnt/β-catenin signaling pathway in zebrafish embryos. This notion is supported by several lines of experimental evidence. First, forced expression of *rspo3* resulted in dorsoanteriorized embryos, resembling those affected by the loss of *wnt3a* and/or *wnt8* [10], [36] and the overexpression of Wnt inhibitors, e.g., *frzb* or *dkk1* [15], [41], [42], [53]. When co-overexpressed with Wnt3a, Rspo3 abolished the exogenous Wnt3a-induced Topflash reporter expression. Overexpression of *rspo3* also inhibited the basal Wnt signaling activity. Second, knockdown of *rspo3* resulted in ventral-posterior phenotype, which resembles those seen in the *dkk1* knockdown embryos [44]. Importantly, knockdown of *rspo3* increased the Wnt reporter activity in a dose-dependent manner and increased the expression of *vent* and *sp5l*. In zebrafish embryos, the zygotic Wnt/β-catenin signaling plays a prominent role in the anterior-posterior neuroectoderm patterning. Our data showed that knockdown of *rspo3* caused neuroectodermal posteriorization, while *rspo3* overexpression led to neuroectodermal anteriorization. These findings strongly support the notion that Rspo3 inhibits the zygotic Wnt/β-catenin signaling. It has been well documented that maternal and zygotic Wnt/β-catenin signaling play opposite roles during zebrafish embryogenesis [1], [4]–[7]. While the maternal β-catenin regulates the formation of the dorsal organizer before gastrulation, the zygotic Wnt/β-catenin signaling initiates ventral cell fates after gastrulation [8], [9]. We tested the possibility that Rspo3 may promote maternal Wnt signaling in zebrafish embryos. This hypothesis was not supported by the results. We found that forced expression of *rspo3* did not affect the expression of *boz* and *chd*. Knockdown of *rspo3* did not decrease *boz* and *chd* expression. In fact, MO1 injection actually increased their expression. Instead, our results suggest that Rspo3 does not promote maternal Wnt signaling in zebrafish embryos.

The finding that Rspo3 inhibits Wnt signaling in zebrafish embryos is unexpected. Several *in vitro* and *in vivo* studies using other model organisms suggest that RSPOs enhance the canonical Wnt signaling by interacting with Lgr4/5/6 and ZNRF3 [18], [19], [21], [54], [55]. The exact reason(s) underlying the different roles of Rspo3 observed in zebrafish compared with mice and *Xenopus* are not clear at present, but there are several plausible explanations. Unlike its mammalian and amphibian counterparts, zebrafish Rspo3 contains three FU domains. This additional FU3 domain is present in all five ray-finned fish species studied but not in the elephant shark, suggesting the FU3 domain is a structural feature that evolved and was conserved in the ray-finned fish lineage. The FU domains are known to be indispensable for mammalian RSPO3/Rspo3 protein activity [22], [25]. A recent report demonstrated that the FU1 and FU2 domains of human RSPO1 are involved in its binding to ZNRF3 and LGR4, respectively [56]. We therefore speculated that its inhibitory role might be attributable to the unique 3 FU structure. This idea, however, was not supported because human RSPO3 had an inhibitory effect similar to that of zebrafish Rspo3 when tested in zebrafish embryos. This finding also indicated that the context of the zebrafish embryo is critical. In zebrafish embryos, *rspo3* mRNA is maternally deposited and has a ubiquitous expression from the 1-cell stage to 12 hpf. After that, however, it displays a tissue-specific expression pattern. This expression pattern in zebrafish differs considerably from those reported in *Xenopus* and mice. In *Xenopus* and mice, the expression of *Rspo3* mRNA is initially detected at the gastrulation stage and in the primitive streak at E7.5, respectively [29], [57]. Future studies will be needed to determine whether the different roles of Rspo3/RSPO3 are related to the different spatial and temporal expression patterns among these different model organisms. It should be mentioned that our *rspo3* mRNA expression result is different from the expression pattern shown in the zebrafish resource ZFIN, which shows no maternal expression and a restricted expression in the forebrain at the 12 hpf stage. An important difference between these two studies lies in the probes utilized. While a 569 bp partial ORF region (95–663 nt) was used in the study deposited in the ZFIN database, our probe covers a 482 bp 3′-UTR sequence plus 322 bp ORF region (633–1436 nt). It is plausible that the longer probe containing a UTR region is more sensitive and has greater specificity. It is noted that Rspo3 morphants had a shorter body axis and a marked lateral expansion of the *myoD* expression domains, suggesting Wnt/PCP signaling may be altered. Studies in *Xenopus* suggest that Rspo3 alter both Wnt/β-catenin and Wnt/PCP signaling [58]. In addition, Wnt/PCP signaling also inhibits the canonical Wnt pathway [59]. It remains to be determined whether Rspo3 indeed alters the Wnt/PCP signaling and whether this possible regulation inhibits the Wnt/β-catenin signaling pathway in zebrafish.

In addition to the Wnt/β-catenin signaling pathway, the Fgf and Nodal signaling pathways are also implicated in the dorsoventral and anterior-posterior patterning in zebrafish embryos [49]–[51]. The Nodal ligand *sqt* gene is expressed in a dorsal region of the blastula and forced expression of *sqt* resulted in expanded or ectopic dorsal mesoderm [49]. Overexpression of *fgf3* dorsalizes zebrafish embryos [50]. The *fgf8* lost-of-function mutant *acerebellar* displayed mild dorsoventral patterning defects [51]. Rspo2, a member of the Rspo family, has been shown to inhibit Nodal signaling in *Xenopus* [57]. We found that overexpression or knockdown of *rspo3* had no significant effect on the mRNA levels of the Nodal ligand *sqt*. The effects of *rspo3* on the Fgf ligands are more complicated. Knockdown of *rspo3* by both MO1 and MO2 increased the expression of *fgf3* mRNA. In the case of *fgf8,* both MO1 and MO2 injected embryos had reduced *fgf8* mRNA levels. However, overexpression of *rspo3* had little effect on the *fgf3* and *fgf8* mRNA levels. In zebrafish, overexpression of *mkp3* ventralizes while knockdown of *mkp3* dorsalizes embryos [52]. Knockdown of *rspo3* has little effect on the expression of *mkp3* mRNA but overexpression of *rspo3* decreases its expression. These changes in Fgf ligands and *mpk3* expression cannot explain the phenotypic changes observed in the *rspo3* overexpression or knockdown embryos.

In summary, ray-finned fish Rspo3 has a unique structural feature and Rspo3 plays an important role in regulating dorsoventral and anterior-posterior patterning in zebrafish embryos. We have provided evidence suggesting that Rspo3 plays a negative role in regulating Wnt/β-catenin signaling in zebrafish embryos. During the revision of this manuscript, Wu et al. (2014) reported that human RSPO2, another member of the RSPO family, plays an inhibitory effect on Wnt/β-catenin signaling in colorectal cancer cells [60]. These new studies suggest that the roles of Rspo/RSPO proteins in the Wnt/β-catenin signaling pathway may be more complex. Future studies will elucidate the molecular mechanisms underlying the inhibitory role of Rspo3/RSPO3 in regulating Wnt/β-catenin signaling. These studies will provide novel insights into Wnt/β-catenin signaling in vertebrates.

## Supporting Information

Table S1 **Primers used in this study.**
(DOCX)
