# Supplementary material for: R-Spondin 3 Regulates Dorsoventral and Anteroposterior Patterning by Antagonizing Wnt/β-Catenin Signaling in Zebrafish Embryos
Source: PLoS One. 2014 Jun 11;9(6):e99514. doi: 10.1371/journal.pone.0099514 (PMC4053527; doi:10.1371/journal.pone.0099514)
Supplement: Table S1 — Primers used in this study. (DOCX) [file pone.0099514.s001.docx]

| name | sequence(5'-3') |
| --- | --- |
| rspo3-F1 | AACCTGTGGCTTCAAATGG |
| rspo3-R1 | TTGTTGTCGCTCATCCAGTA |
| beta-actin-F | CTTGCGGTATCCACGAGAC |
| beta-actin-R | GCGCCATACAGAGCAGAA |
| qboz-F | GATGTACTGCTGCTGCGTTCC |
| qboz-R | CTGCTCCGTCTGGTTGTCG |
| qvent-F | GGAGAGTGATGACAGTGAAGTAGA |
| qvent-R | ACAGCGGGATAGAGGAAGT |
| qsp5l-F | GGAGGTCACGTTGAGGATGG |
| qsp5l-R | GCGACAGCGACGAGTAGAGC |
| qfgf3-F^*^ | GTGGCAATCAAGGGACTGTT |
| qfgf3-R^*^ | GCCGTGATGCATAAGTGTTG |
| qfgf8-F^*^ | AATCGCAGAGCACAGACCCTT |
| qfgf8-R^*^ | GGCTTTCGGTCCTCTCCTTTT |
| qmkp3-F^*^ | TACTGCTGAGGAGAATGAA |
| qmkp3-R^*^ | ACTGCTGCTTGACGAACCGT |
| qsqt-F^*^ | TCGGTGTGAAGGCAGTTGTC |
| qsqt-R^*^ | GTACGCAGGACAAGCATGG |
| qchd-F^*^ | ACGCCTGCTGCCATACAAT |
| qchd-R^*^ | CACTGAGGGTCCACCGAGA |
| qbeta-actin-F | ACAGGGAAAAGATGACACAG |
| qbeta-actin-R | AGAGTCCATCACGATACCAG |
| rspo3 probe-F | AACCTGTGGCTTCAAATGG |
| rspo3 probe-R | TTGTTGTCGCTCATCCAGTA |

*, fgf3 sequence from ”Dynamic Expression of FGF3 Gene during Zebrafish Embryonic Development.” Jia Congcong et al., Chinese Journal of Cell Biology 2012, 34(4):355–360.

fgf8 sequence from ” Mutation of the atrophin2 gene in the zebrafish disrupts signaling by fibroblast growth factor during development of the inner ear.” Yukako Asai et al., Proc Natl Acad Sci 2006, 103(24):9069-74.

makp3 sequence from “A novel chemical screening strategy in zebrafish identifies common pathways in embryogenesis and rhabdomyosarcoma development.” Xiuning Le et al., Development 2013, 140(11):2354-64.

sqt sequence from “Activin-betaA Signaling Is Required for Zebrafish Fin Regeneration.” Anna Jaźwińska et al., Curr. Biol. 2007, 17(16):1390-1395.

chd sequence from “IGF binding protein 3 exerts its ligand-independent action by antagonizing BMP in zebrafish embryos.” Yingbin Zhong et al., J Cell Sci. 2011, 124(Pt 11):1925-35.
